# Coupled Biphasic NaMnO_2_ Cathode and Ti_3_C_2_ MXene Anode with Complementary Charge-Storage Kinetics for Aqueous and Non-aqueous Sodium-Ion Hybrid Energy Storage

**DOI:** 10.1021/acs.energyfuels.6c00118

**Published:** 2026-03-13

**Authors:** Tetiana Boichuk, Andrii Boichuk, Mahesh Eledath Changarath, João Fonseca, Marie Krečmarová, Saïd Agouram, Maria C. Asensio, Juan F. Sánchez-Royo

**Affiliations:** † 16781 Instituto de Ciencia de los Materiales de la Universidad de Valencia (ICMUV), 46071 Valencia, Spain; ‡ King Danylo University, 76000 Ivano-Frankivsk, Ukraine; § Department of Applied Physics and Electromagnetism, University of Valencia, 46100 Valencia, Spain; ∥ Instituto de Ciencia de Materiales de Madrid (ICMM), Consejo Superior de Investigaciones Científicas (CSIC), 28049 Madrid, Spain; ⊥ MATINÉE: CSIC Associated Unit between the Instituto de Ciencia de los Materiales de la Universidad de Valencia (ICMUV) and the ICMM, Cantoblanco, 28049 Madrid, Spain

## Abstract

A metallic sodium-free hybrid electrochemical system has been developed by coupling a biphasic birnessite-type NaMnO_2_ cathode with a multilayer Ti_3_C_2_T_*x*_ MXene anode, exhibiting complementary and cooperative electrochemical behavior. The orthorhombic/monoclinic NaMnO_2_ structure enables stable Na^+^ intercalation/deintercalation, while Ti_3_C_2_T_*x*_ provides fast, surface-driven pseudocapacitive charge storage with favorable interfacial kinetics. This combination offers kinetic complementarity between diffusion-controlled and surface-dominated processes, leading to efficient charge balancing and enhanced rate performance. Assembled coin cell devices exhibit outstanding rate capability and cycling stability in both aqueous (Na_2_SO_4_) and non-aqueous (NaPF_6_ in EC/DMC) electrolytes with a higher voltage window for enhancing energy density. Energy and power densities reach 90 and 610 W/kg in the aqueous system, and 360 and 970 W/kg in the organic one, with 97 and 88% capacity retention after 1000 cycles, respectively. The demonstrated universality of this electrode pairing establishes a versatile, safe, and sustainable strategy for high-performance sodium-ion energy storage beyond metallic sodium systems.

## Graphical abstract

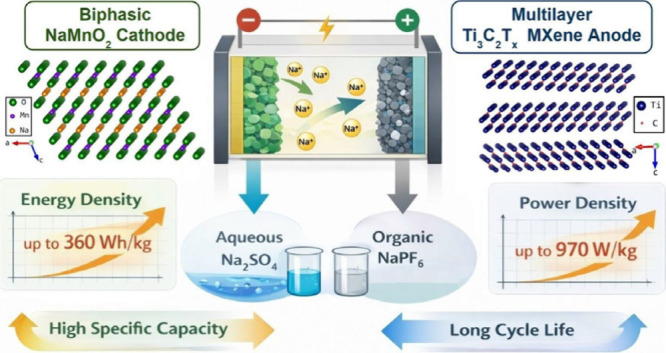

## Introduction

The growing demand for large-scale energy storage as well as limited lithium supply and escalating costs has brought renewed attention to alternatives beyond conventional lithium-ion power sources.^1–3^ Sodium technology is the closest option with similar operational principles and redox chemistry for the development of power sources,^4–7^ such as conventional sodium-ion batteries,^7–12^ supercapacitors,^13–16^ and safer and sustainable metal-free power sources.^17,18^ Avoiding the use of metallic sodium can significantly reduce the overall cost of commercial devices and minimize the risks of explosion, dendrite formation, and instability in common electrolytes, which can lead to short-circuiting and poor cycle life. Moreover, it may be possible to enhance electrochemical performance due to cooperative effects between the pseudocapacitive materials of the electrodes.

Recent strategies for designing cathode/anode combinations focus on achieving compatibility in their charge storage mechanisms (particularly pseudocapacitance) across materials with different electrochemical potentials. With the pairing of electrodes that exhibit complementary pseudocapacitive behavior, it becomes possible to ensure rapid and reversible charge accumulation in both electrodes, even at high current densities. This compatibility not only enhances the power performance of the electrochemical system but also broadens the operating potential window, increasing the overall energy density. Layered presodiated birnessite-type NaMnO_2_ is particularly attractive because of its flexible framework and ability to host sodium ions reversibly.^19–22^ Sol–gel synthesized biphasic NaMnO_2_, as demonstrated in our previous publications,^23,24^ consists of orthorhombic and monoclinic domains that enhance structural stability and Na-ion transport. Due to its relatively small particle size and optimized morphology, biphasic NaMnO_2_ as a cathode material delivers a specific capacity over 100 mAh/g in an aqueous sodium electrolyte during long-term cycling.^23^ Moreover, even at high current densities, the material exhibits good structural stability with an optimized diffusion/capacity ratio.^24^ These features make this type of biphasic layered manganese oxide suitable for a wide range of aqueous and non-aqueous electrochemical systems, such as batteries and hybrid supercapacitors.

MXenes, as a new family of 2D materials,^25,26^ especially Ti_3_C_2_, have emerged as promising candidates for an anode.^27–31^ Considering two commonly used electrode states of MXene, single flakes and multilayered (ML) MXene, despite the lower conductivity, ML MXenes^32^ are more suitable for high-rate electrodes. Particularly, as we have shown in ref 33, ML Ti_3_C_2_T_*x*_ exhibits excellent cycling stability in a Na_2_SO_4_ electrolyte, delivering a specific capacity of about 112 mAh/g at high scan rates, with classical pseudocapacitive behavior and a predominance of a capacitive-controlled charge accumulation mechanism. With the coupling of both materials in a single electrochemical system, the diffusion-controlled Na^+^ intercalation of the NaMnO_2_ cathode contrasts with the fast pseudocapacitive charge storage of the MXene anode, enabling complementary kinetics that alleviate rate limitations in the full cell.

Guided by the metallic sodium-free design concept and the complementary electrochemical behavior of orthorhombic/monoclinic NaMnO_2_ and multilayer MXene Ti_3_C_2_T_*x*_, this work presents a high-performance sodium-based hybrid system combining these materials within a single cell. Unlike previously reported sodium-ion or hybrid systems, this work demonstrates a sodium metal-free electrochemical system that combines diffusion-controlled biphasic NaMnO_2_ and surface-dominated Ti_3_C_2_T_*x*_ to exploit kinetic complementarity, balanced charge storage, high-rate performance, and long-term stability. Its efficient operation in both aqueous and non-aqueous electrolytes highlights a versatile and practical strategy for next-generation sodium-based energy storage, establishing a new design pathway for safe, low-cost, and sustainable Na metal-free technologies.

## Experimental Section

### Synthesis of Electrode Materials

Biphasic sodium manganese oxide NaMnO_2_ has been prepared using the sol–gel method, following the procedure reported earlier.^23^ A 0.2 M aqueous solution of manganese acetate tetrahydrate and sodium nitrate was stirred at room temperature for 5 h. The pH was adjusted to 8 using a 25% ammonia solution, followed by being in a vacuum oven for 24 h at 80 °C. The resulting product was ground, annealed at 750 °C for 15 h (heating rate of 3 °C min^–1^), and then allowed to cool naturally to room temperature. MAX phase Ti_3_AlC_2_ (Figure S1) was synthesized by solid-state reaction^33^ using commercial Ti, Al metallic powders, and graphite as starting precursors. The MAX etching procedure using HF/HCl solution^34^ was conducted at 35 °C for 48 h with further centrifugation (6 cycles, 2700 rpm, and 8 min each cycle), washing with deionized (DI) water and vacuum filtration in order to obtain ML Ti_3_C_2_T_*x*_ powder. The synthesis schemes of the electrode materials are presented in Figure 1a (NaMnO_2_) and Figure 1b (Ti_3_C_2_T_*x*_).

**Figure 1:**
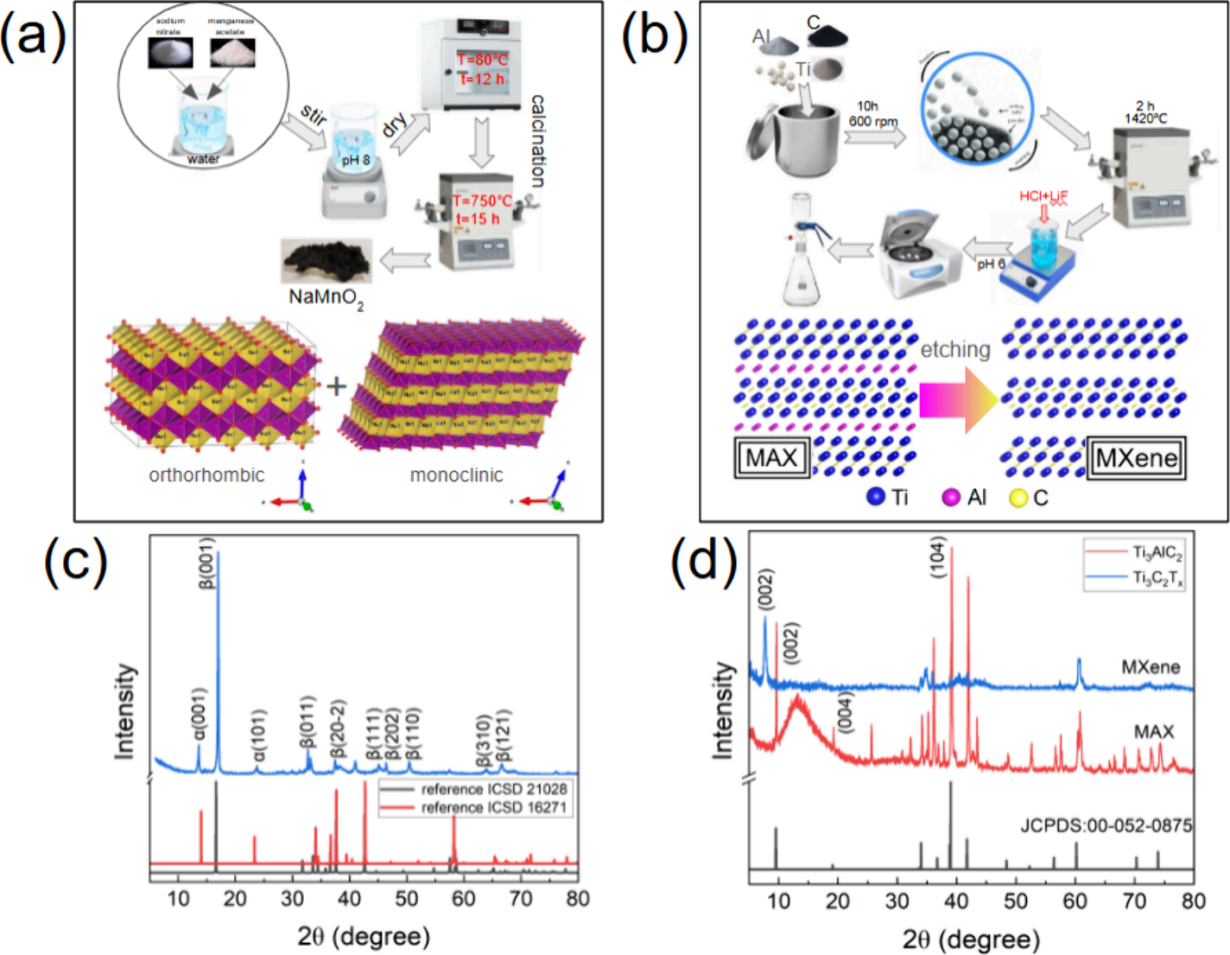
Schematic illustration and structural characterization of the synthesized electrode materials: (a) synthesis route for biphasic NaMnO_2_ (sol–gel synthesis) and (b) preparation process of multilayer Ti_3_C_2_T_*x*_ MXene, including ball milling, calcination, etching and vacuum filtration of ML MXene, (c) XRD pattern of biphasic orthorhombic/monoclinic NaMnO_2_ compared to the reference, and (d) XRD patterns of the Ti_3_AlC_2_ MAX phase and obtained Ti_3_C_2_T_*x*_, indicating successful Al removal and an increase of the interlayer distance.

### Characterization Methods

X-ray diffraction (XRD) patterns were recorded using a Bruker D8 ADVANCE A25 diffractometer with Cu Kα radiation (λ = 1.54 Å) in the 2θ range of 5–80° and a step size of 0.02°. The surface morphology of the samples was examined by field emission scanning electron microscopy (FESEM, SCIOS 2, 10 kV). High-resolution transmission electron microscopy (HRTEM) was conducted by using a TECNAI G2 F20 microscope operating at 200 kV. X-ray photoelectron spectroscopy (XPS) measurements were performed using a Thermo Scientific K-Alpha system with monochromatic Al Kα radiation (1486.6 eV and base pressure of ∼1 × 10^–10^ mbar) to analyze surface composition and oxidation states.

### Electrochemical Measurement Technology

The electrochemical performance was investigated by using a coin cell (CR2032). Multilayer MXene powder (anode) and NaMnO_2_ (cathode) as the active materials of electrodes (80 wt %) were ground with 15 wt % conductive carbon black (CB) and 5 wt % PTFE binder in a mortar. The mass loading of each negative and positive electrode (diameter of both was 11 mm) was calculated and used to balance the charge between the two electrodes (see the Supporting Information) based on measurements in a three-electrode cell with a Ag/AgCl reference electrode. In particular, active material loading was 3.16 mg/cm^2^ for MXene-based electrodes and 4.65 mg/cm^2^ for the cathode based on NaMnO_2_. A cyclic voltammetry (CVA) test was conducted (Gamry 5000E equipment) in an aqueous 1 M Na_2_SO_4_ and non-aqueous NaPF_6_ (EC/DMC) solution (scan rates of 0.5–50 mV/s). Based on CV at different scan rates, capacitive/diffusion contribution has been calculated based on the current response (*I*) at a fixed voltage (*V*) based on the following equation:^23,24,35^

1 $$I\left(V\right)=k_{1}v+k_{2}v^{1/2}$$

where *v* is the scan rate, *k*_1_ is the capacitive contribution, and *k*_2_ is the diffusion contribution. Galvanostatic charge–discharge cycling was performed using ARBIN LBT21084 at room temperature with a voltage window of 0–1 V (Na_2_SO_4_ electrolyte) and 0–3 V (NaPF_6_ electrolyte). Values of specific capacitance can be achieved using the cyclic voltammogram as follows:

2 C=S/(2ΔUmv)

Based on galvanostatic charge–discharge measurements, the specific capacity *Q*, energy density *E*, and power density *P* were calculated using the following equations:

3 Q=IΔt/3.6m

4 $$E=0.5C\Delta U^{2}/3.6$$

5 P=(3600E)/Δt

where *C* is the specific capacitance (F/g), *Q* is the specific capacity (mAh/g), *S* is the integrated area of the CV curve, *m* is the mass of active materials from both the cathode and anode material, *v* is the scan rate, *I* is the applied discharge current, Δ*U* is the operating voltage window, and Δ*t* is the discharge time.

Electrochemical impedance spectroscopy (EIS) was performed using Gamry 5000E equipment before cycling, after 1 cycle, and after 1000 charge–discharge cycles. Measurements were conducted at open-circuit voltage in the frequency range from 20 kHz to 0.01 Hz (63 points), with the amplitude of the alternating current voltage being 5 mV.

## Results and Discussion

Temperature/time conditions of NaMnO_2_ synthesis and final calcination in air (Figure 1a) allowed us to obtain a biphasic composite. The XRD pattern of NaMnO_2_ (Figure 1c) displays distinct reflections corresponding to both orthorhombic (β-NaMnO_2_, space group *Pmmn*) and monoclinic (α-NaMnO_2_, space group *C*2/*m*) phases, with a rough phase distribution of about 40:60%. The presence of the (001), (002), and (111) reflections characteristic of β-NaMnO_2_, together with the (011) and (112) reflections from α-NaMnO_2_, verifies the coexistence of the two structural motifs. The orthorhombic phase features an ordered zigzag arrangement of MnO_6_ octahedra, while the monoclinic phase exhibits a distorted framework with alternating edge- and corner-sharing octahedra. As reported in refs 23 and 24 , coexistence of these two polymorphs is known to generate interfacial strain and structural disorder, which can facilitate Na^+^ migration through interlayer channels and enhance structural stability during cycling and shows good electrochemical performance in sodium electrolytes. In particular, the monoclinic phase facilitates fast Na ion diffusion, supporting high rate capability, while the orthorhombic phase provides a more rigid lattice that enhances the structural stability and ensures long-term cycling performance. The Ti–Al–C compound (Figure 1d) has a strong peak of Ti_3_AlC_2_ MAX phase at ≈9.51° [(002) plane in a hexagonal structure]. After HF/HCl etching, we observe a noticeable shift of the (002) peak toward lower 2θ angles (about 7.73°). The disappearance of the (104) and (105) peaks associated with the Ti_3_AlC_2_ MAX phase confirms complete Al extraction. The calculated interplanar distance for Ti_3_AlC_2_ is about 0.94 and 1.15 nm for ML MXene, obtained after etching, centrifugation, washing, and filtration. Successful Al removal and an increase in interlayer spacing due to surface termination groups (−O, −OH, and −F) play a critical role in surface charge regulation and interlayer spacing, which influence both hydrophilicity and ion intercalation capability.

SEM image of NaMnO_2_ (Figure 2a) depicts agglomerated platelet-like particles usually having sizes that are within the sub-micrometer to micrometer range, consistent with the layered presodiated manganese oxide sheets. The TEM image (Figure 2b) demonstrates clearly defined lattice fringes related to orthorhombic and monoclinic phases, confirming the biphasic structure of NaMnO_2_ and indicating well-ordered atomic planes (Table ST1).

**Figure 2:**
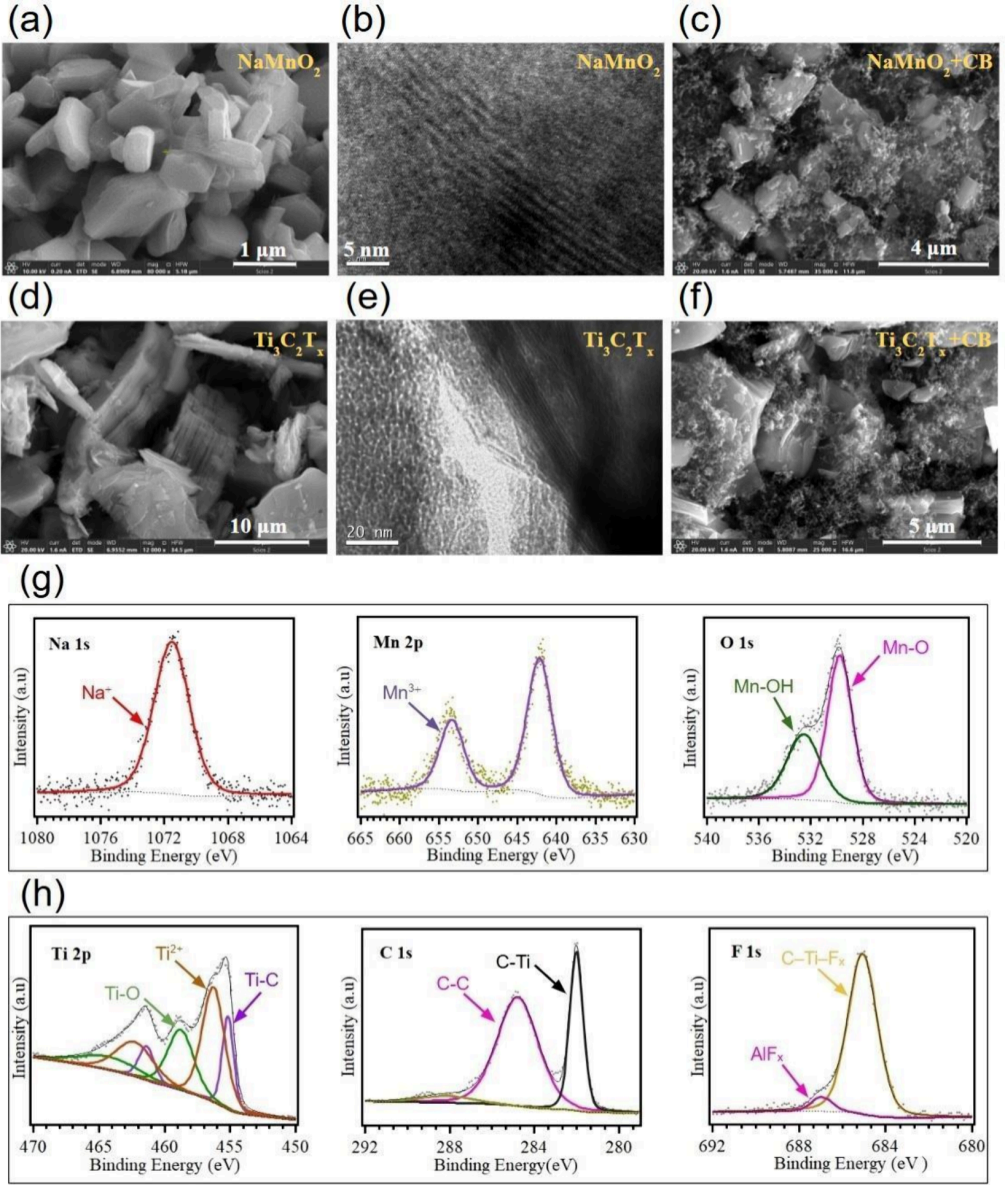
Morphology and composition of biphasic NaMnO_2_ and Ti_3_C_2_T_*x*_ ML MXene: (a) SEM of biphasic layered NaMnO_2_ with a uniform distribution of particles, (b) TEM of NaMnO_2_ particles showing a layered structure, (c) SEM of the cathode composition NaMnO_2_/CB, (d and e) Ti_3_C_2_T_*x*_ microstructure as a demonstration of the interlayer distance increasing, (f) SEM of the ML MXene/CB anode composite, (g) XPS spectra of NaMnO_2_, and (h) XPS of Ti_3_C_2_T_*x*_ with the corresponding components.

The pristine Ti_3_C_2_T_*x*_ MXene exhibits a typical accordion-like morphology (Figure 2d) with an average particle size of 10 μm, characteristic of multilayered structures resulting from the selective etching of the Al layer from the MAX phase. The TEM image (Figure 2e) further confirms the layered configuration of the MXene sheets with an interlayer spacing of about 1.1 nm, indicating successful exfoliation and preservation of the two-dimensional structure. Carbon black particles (conductive additive that we added for electrode preparation) are observed to be uniformly distributed across the NaMnO_2_ particles (Figure 2e) and ML MXene (Figure 2e), forming a conductive composite network based on EDS of composites with CB (Figure S2)

X-ray photoelectron spectroscopy (XPS) was conducted to analyze the chemical states of the elements in both NaMnO_2_ and MXene samples. Figure 2g displays the Na 1s, Mn 2p, and O 1s core-level spectra obtained from the NaMnO_2_ sample, while Figure 2h presents the Ti 2p, C 1s, and F 1s core-level spectra acquired from the MXene sample. The spectra were deconvoluted by assuming a Voigt line shape and Shirley backgrounds. The Na 1s spectrum exhibits a single peak at 1071.6 eV, corresponding to Na^+^ ions within the NaMnO_2_ lattice. The Mn 2p spectrum displays a characteristic doublet, with the 2p_3/2_ peak centered at 642.2 eV, attributed to Mn^3+^ ions in NaMnO_2_.^38^ Deconvolution of the O 1s spectrum reveals two distinct components, assigned to Mn–O bonds (529.75 eV) and surface hydroxyl groups (532.6 eV).^39^ In the Ti 2p spectrum, deconvolution identifies three separate doublets, corresponding to Ti–C (455.1 eV), Ti^2+^ (456.2 eV), and TiO_2_ (458.7 eV).^36^ The C 1s spectrum contains three distinct peaks, assigned to Ti–C (282 eV), C–C (284.8 eV), and C=O (288.1 eV) bonding states.^36,37^ The F 1s spectrum shows two prominent peaks attributed to C–Ti–F_*x*_ (685 eV) and AlF_*x*_ (686.8 eV) surface groups. A low-intensity AlF_*x*_ component is commonly observed in multilayered MXenes^40,41^ and does not indicate unsuccessful etching. The absence of Al peaks in the survey XPS spectra (Figure S3) confirms effective removal of Al and suggests that the AlF_*x*_ signal arises from strong Al–F interactions, likely due to incomplete washing. Given its very low intensity and the fact that fluorine is present only as surface terminations, this component is not expected to affect the electrochemical properties of the MXenes. The Cl 2p spectrum was also obtained with the detected signal attributed to residual chlorine originating from the HCl etching process; this spectrum is provided in Figure S4.

To investigate the electrochemical properties of NaMnO_2_ and ML MXene separately, we performed cyclic voltammetry (CVA) measurements of both electrodes at different scan rates using a three-electrode cell in an aqueous electrolyte (1 M aqueous solution of Na_2_SO_4_). The NaMnO_2_-based electrode operates at higher potentials (from −0.25 to 0.8 V vs Ag/AgCl) compared to the MXene-based electrode (Figure 3a). At low scan rates (Figure S5), both materials exhibit pseudocapacitive behavior, showing similar enclosed areas of the CVA curves and distinct cathodic and anodic peaks. At a scan rate of 50 mV/s (Figure 3a), the MXene-based electrode shows a higher capacity due to the predominance of a surface-controlled pseudocapacitive charge accumulation mechanism. Since the NaMnO_2_-based electrode has a smaller surface area, the ratio between capacitive and diffusion-controlled mechanisms is lower, resulting in larger reduced specific capacity at high scan rates vs MXene (Figure S6) but enhanced long-term stability, as shown in ref 24. Based on these CVA results, it is promising to investigate the pairing of these two electrodes in commercial coin cells to evaluate their overall electrochemical performance.

**Figure 3:**
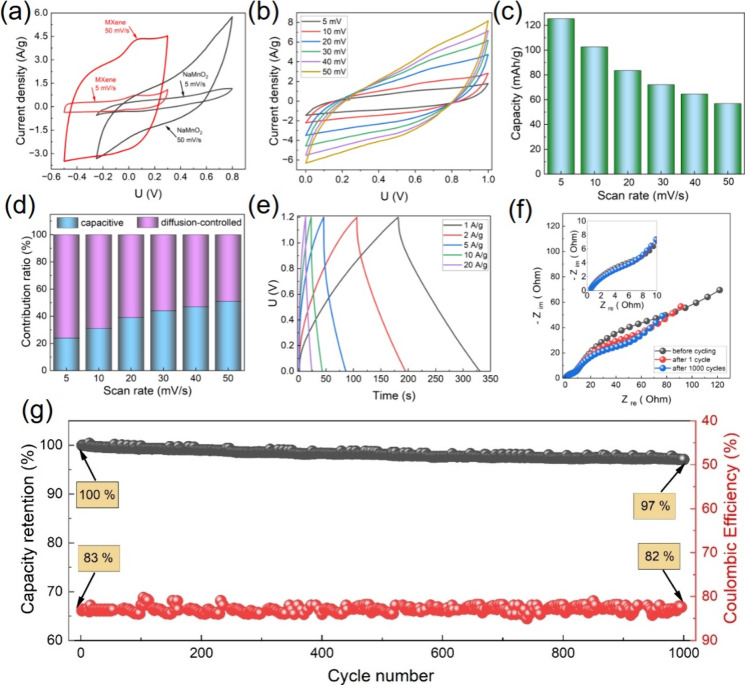
Electrochemical performance of the NaMnO_2_/Ti_3_C_2_T_*x*_ electrode combination in an aqueous Na_2_SO_4_ electrolyte: (a) CVA curves of NaMnO_2_ and Ti_3_C_2_T_*x*_ in a three-electrode cell, (b) CVA curves of the coin cell with the NaMnO_2_ cathode and Ti_3_C_2_T_*x*_ at different scan rates, (c) specific capacity of the coin cell at various scan rates, (d) quantitative analysis of capacitive and diffusion-controlled contributions at different scan rates, (e) galvanostatic charge–discharge curves at different current densities, (f) Nyquist plots of the coin cell before cycling, after first cycle, and after 1000 cycles, and (g) long-term cycling performance and coulombic efficiency of the coin cell.

CVA of manufactured coin cell CR2032 based on the NaMnO_2_ cathode and ML Ti_3_C_2_T_*x*_ anode in a Na_2_SO_4_ electrolyte shows pseudocapacitive behavior (Figure 3b) at different scan rates with low-intensity cathodic and anodic peaks and good stability during 1000 CVA cycles at 50 mV/s (Figure S7). The quasi-rectangular shapes and symmetric redox features suggest stable and reversible charge accumulation with mixed capacitive and diffusion contributions with a good rate response. The capacity decreases gradually with an increasing scan rate (Figure 3c) due to kinetic limitations and ion diffusion resistance at higher rates, mostly on the core of NaMnO_2_. Particularly, as we have shown,^23,24^ biphasic presodiated manganese oxide in an aqueous electrolyte provides intercalation/deintercalation of Na^+^ as well as insertion of OH^–^ groups due to the formation of a birnessite-like structure, providing a high contribution of diffusion-controlled pseudocapacitance at low scan rates.

On the contrary, the capacitive contribution increases with the scan rate (Figure 3d), demonstrating the predominant pseudocapacitive charge accumulation on the surface of the MXene electrode that enhances high-rate capability. Galvanostatic charge–discharge profiles at different current densities (Figure 3e) exhibit quasi-linear sloping voltage curves, characteristic of a hybrid charge storage mechanism that combines faradaic redox reactions at the cathode with surface-controlled capacitive behavior at the anode. The absence of distinct voltage plateaus and the symmetric nature of the charge–discharge profiles indicate rapid and reversible insertion/extraction kinetics and minimal polarization, highlighting the excellent rate capability of the coin cell with this combination of electrode materials. Nyquist plots obtained before and during cycling reveal a small semicircle in the high- and medium-frequency region and a line in the low-frequency region (Figure 3f). Semicircles correspond to low solid/electrolyte interphase (SEI) and charge-transfer resistance, which decrease from 42 Ω before cycling to 28 Ω after 1000 cycles, attributed to the formation of a stable and ion-permeable SEI layer during cycling^42–44^ as well as improved electrolyte wetting, and interfacial stabilization enhances charge-transfer kinetics at the electrode–electrolyte interface. A gradual increase in double-layer capacitance *C*_dl_ from 38 μF before cycling to 55 μF after cycling and the linear low-frequency part indicate efficient ion diffusion within the electrode/electrolyte interface,^45,46^ suggesting an enhanced electrochemically active surface area and improved electrolyte–electrode contact. Long-term cycling stability and coulombic efficiency over 1000 cycles demonstrate 97% capacity retention and ∼82% coulombic efficiency (Figure 3g). The relatively low coulombic efficiency (∼82%) in the aqueous full cell arises from water-induced side reactions in the Na_2_SO_4_ electrolyte, including hydrogen/oxygen evolution and interfacial reactions that consume charge irreversibly.

The combination of the NaMnO_2_ cathode and Ti_3_C_2_T_*x*_ anode (CR2032 coin cells) in an organic electrolyte (NaPF_6_ in EC/DMC) exhibits distinct electrochemical performance compared to results in an aqueous electrolyte. As shown in Figure 4a, the CVA curves maintain quasi-rectangular shapes with visible redox peaks, reflecting a balanced hybrid energy storage mechanism combining the pseudocapacitive behavior of Ti_3_C_2_T_*x*_ and faradaic intercalation/deintercalation in NaMnO_2_. Figure 4b shows a higher capacitive contribution than that in the aqueous system. One of the reasons is a wider working voltage window (0–3 V), which enables rapid surface-controlled charge storage on Ti_3_C_2_T_*x*_ and stable interfacial behavior. Despite weak Na^+^ solvation and improved surface stability of MXene, fast ion adsorption and desorption are favored in the aqueous system due to strong hydration and a narrow voltage range. The charge–discharge curves (Figure 4c) show smooth slopes with a high coulombic efficiency, indicating efficient Na^+^ transport and stable electrode kinetics. The EIS spectra (Figure 4d) display a higher overall impedance than in the aqueous system, with two high/medium-frequency semicircles, indicating the presence of two separate interfacial processes. The small semicircle at high frequency corresponds to the resistance and capacitance of the SEI film that forms on the surface of electrodes during the initial cycles and does not change a lot during cycling. The second, larger semicircle in the medium-frequency region is associated with the charge transfer process at the electrode/electrolyte interface.

**Figure 4:**
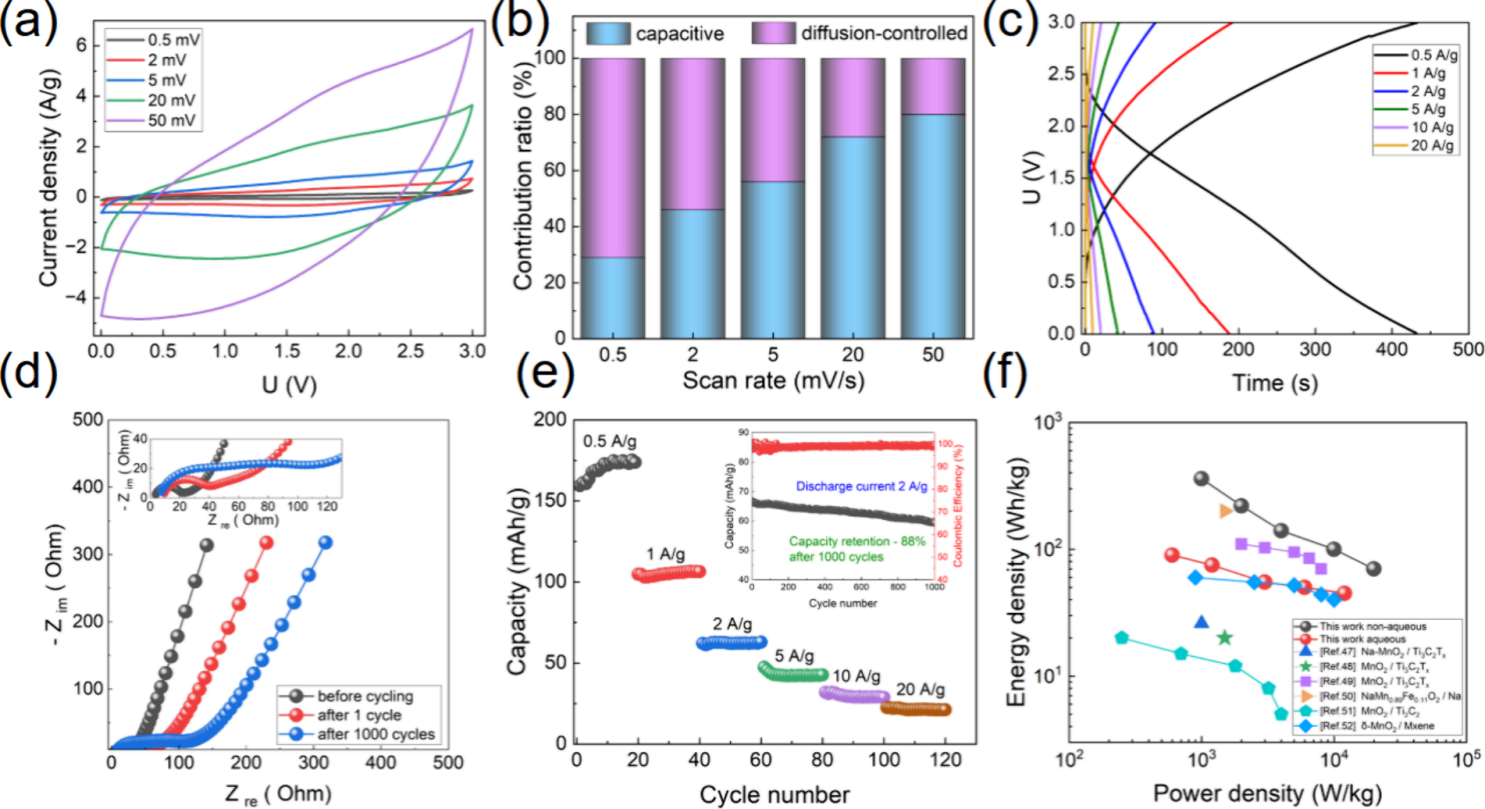
Electrochemical performance of the NaMnO_2_/Ti_3_C_2_T_*x*_ coin cell in a non-aqueous NaPF_6_ electrolyte with organic solvents (EC/DMC): (a) CVA curves at various scan rates, (b) quantitative analysis of capacitive and diffusion-controlled contributions at different scan rates, (c) galvanostatic charge–discharge profiles at different current densities, (d) Nyquist plots before cycling, after the first cycle, and after 1000 charge–discharge cycles, (e) rate performance at varying current densities with an inset showing long-term cycling, and (f) Ragone plot comparing the energy and power densities of this work to previously reported aqueous and non-aqueous systems.

The organic electrolyte shows an increase in interfacial resistance *R*_ct_ up to 120 Ω after 1000 cycles and a corresponding decrease in *C*_dl_ (from 53 to 13 μF after 1000 cycles). In the organic electrolyte, charge–discharge cycling leads to the gradual growth of a passivating SEI layer at the electrode–electrolyte interface. Continuous electrolyte and PF_6_^–^ decomposition results in the formation of a thick and poorly conductive interphase that progressively covers the electrochemically active surface of the electrodes. This passivating film increases the charge-transfer resistance, as reflected by the rise in *R*_ct_ to 120 Ω after 1000 cycles, while simultaneously reducing the effective interfacial area accessible to the electrolyte, leading to a pronounced decrease in the double-layer capacitance. The simultaneous increase in *R*_ct_ and decrease in *C*_dl_ suggest that, in the organic system, the SEI continues to grow during cycling, progressively passivating the electrode surface and limiting Na ion transport across the interface. These impedance trends correlate with the CVA (Figure S8) and long-term charge–discharge results, showing 88% capacity retention after 1000 cycles (Figure 4e, inset). The different electrochemical responses observed in aqueous and organic electrolytes originate primarily from how Na^+^ ions interact with the solvent and electrode surface. In the aqueous system, sodium ions are surrounded by a strong hydration shell, which facilitates rapid ion transport and promotes surface-dominated charge storage on Ti_3_C_2_T_*x*_. At the same time, the narrow stability window of water limits the operating voltage and introduces parasitic reactions that influence the properties (in our case, relatively low coulombic efficiency). In the organic electrolyte, Na^+^ experiences a different solvation environment and operates within a much wider potential window. This enables higher energy output but also leads to the formation of a solid electrolyte interphase, fundamentally changing the nature of the electrode–electrolyte interface and its evolution during cycling. With regard to cycling stability, the coexistence of orthorhombic and monoclinic domains in NaMnO_2_ helps accommodate repeated Na^+^ insertion/extraction without structural degradation. At the same time, the predominantly surface-controlled pseudocapacitive behavior of the MXene anode ensures fast and highly reversible charge storage with minimal diffusion limitations. In an aqueous electrolyte, the interface remains relatively dynamic yet stable over extended operation. In contrast, in the organic system, gradual SEI growth progressively modifies the interfacial resistance, which explains the slight decline in capacity despite good coulombic efficiency.

Based on the performance comparison of aqueous and non-aqueous hybrid systems (Table ST2), despite less stability, batteries in an organic electrolyte provide higher values of specific capacity (about 170 mAh/g) and perfect rate capability with constant coulombic efficiency (Figure 4e), highlighting the robustness of both electrodes. The graph (Figure 4f) compares the energy and power densities calculated in this work to previously reported NaMnO_2_/MXene^47,50^ or (MnO_2_)/MXene^48–52^ combinations for Na-ion batteries, with a detailed description in Table ST3. As a result, biphasic NaMnO_2_/Ti_3_C_2_T_*x*_ hybrid systems demonstrate superior performances in both aqueous (*E* = 90 Wh/kg and *P* = 610 W/kg) and non-aqueous (*E* = 360 Wh/kg and *P* = 970 W/kg) electrolytes, indicating the possibility of using such electrodes in high-energy electrochemical devices.

## Conclusion

This work demonstrates a universal and synergistic electrode design for metallic sodium-free Na-ion hybrid energy storage. The combination of biphasic NaMnO_2_ and multilayer Ti_3_C_2_T_*x*_ MXene enables highly reversible hybrid charge storage, where diffusion-assisted intercalation at the cathode complements surface-controlled pseudocapacitance at the anode. The structural compatibility and stable interfacial behavior between these materials result in fast charge transfer, low polarization, and long-term durability in both aqueous and organic electrolytes. The ability of the same electrode couple to operate efficiently in different electrolyte environments highlights its robustness and practical adaptability. Beyond its excellent electrochemical performance, this approach introduces a new concept for designing safe, low-cost, and high-energy sodium-based systems without metallic sodium, offering a promising direction for large-scale and sustainable energy storage technologies.

## Supplementary Material



## Data Availability

Data will be made available on request.

## Notes

Detailed information about the NaMnO_2_ structure and morphology, SEM images of the MAX phase, XPS survey spectra of Ti_3_C_2_T_*x*_ MXene, methodology of the electrode mass loading calculation, CVA curves at a low scan rate for separated electrodes in a three-electrode cell, capacity retention during the first 50 cycles of separated electrodes, CVA curves of aqueous and non-aqueous hybrid devices during 1000 cycles, and comparison of the characteristics of reported Ragone plot systems (PDF)
