# Supplementary material for: Coupled Biphasic NaMnO2 Cathode and Ti3C2 MXene Anode with Complementary Charge-Storage Kinetics for Aqueous and Non-aqueous Sodium-Ion Hybrid Energy Storage
Source: Energy Fuels. 2026 Mar 13;40(12):6453–61. doi: 10.1021/acs.energyfuels.6c00118 (PMC13040544; doi:10.1021/acs.energyfuels.6c00118)
Supplement: Supplementary file 1 [file ef6c00118_si_001.pdf]

Supplementary information (SI)

Coupled Biphasic $NaMnO_2$ Cathode and Ti_3C_2 MXene Anode with Complementary Charge-Storage Kinetics for Aqueous and Non-Aqueous Sodium-ion Hybrid Energy Storage

Tetiana Boichuk[1*], Andrii Boichuk[1,2*], Mahesh Eledath Changarath[1], João Fonseca[1], Marie Krečmarová[1], Saïd Agouram[3], Maria C. Asensio[4,5], Juan F. Sánchez-Royo[1,5*]

[1] ICMUV, Instituto de Ciencia de Materiales, Universidad de Valencia, 46071 Valencia, Spain

[2] King Danylo University, 76000, Ivano-Frankivsk, Ukraine

[3] Department of Applied Physics and Electromagnetism, University of Valencia, 46100 Valencia

[4] Instituto de Ciencia de Materiales de Madrid ICMM, CSIC, 28049 Madrid, Spain

[5] MATINÉE: CSIC Associated Unit Between the Instituto de Ciencia de los Materiales de la Universidad de Valencia (ICMUV). c/ Catedrático José Beltrán 2, 46980 Paterna (Valencia) and the ICMM, Cantoblanco 28049 Madrid, Spain.

*Corresponding author: Andrii.Boichuk@uv.es (Andrii Boichuk)

*Corresponding author: Tetiana.Boichuk@uv.es (Tetiana Boichuk)

†Corresponding author: Juan.F.Sanchez@uv.es (Juan F. Sánchez-Royo)

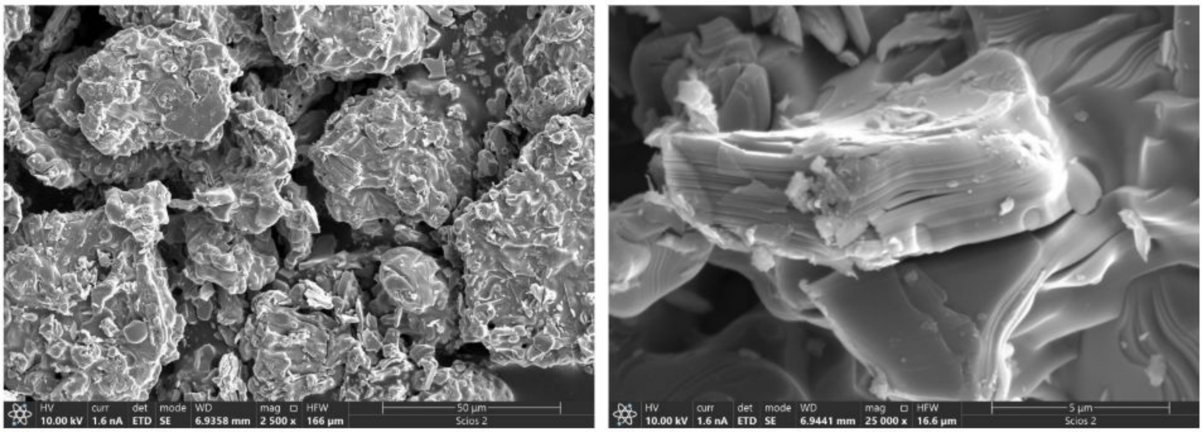

Figure S1. SEM imagenes Max Phase

$NaMnO_2$	
d_{hkl} (nm)	(hkl)/ phases
0.64	(001) ortho
0.55	(001) mono
0.24	(200)/(110)ortho &/or (-111)/(-202) mono
0.21	(111) mono
0.14	(-400)/(-313)/<br>(-204)/(020)/(-402)/<br>(-401) mono

Table ST1. Interplanar distances extracted from SAED patterns for $NaMnO_2$

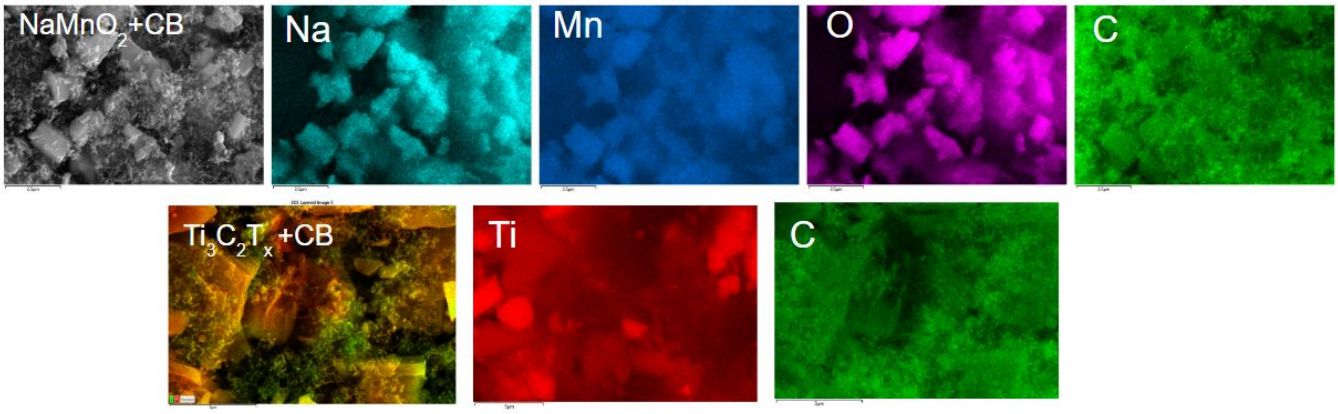

Figure S2. EDS of $NaMnO_2$ and ML Mxene mixed with CB

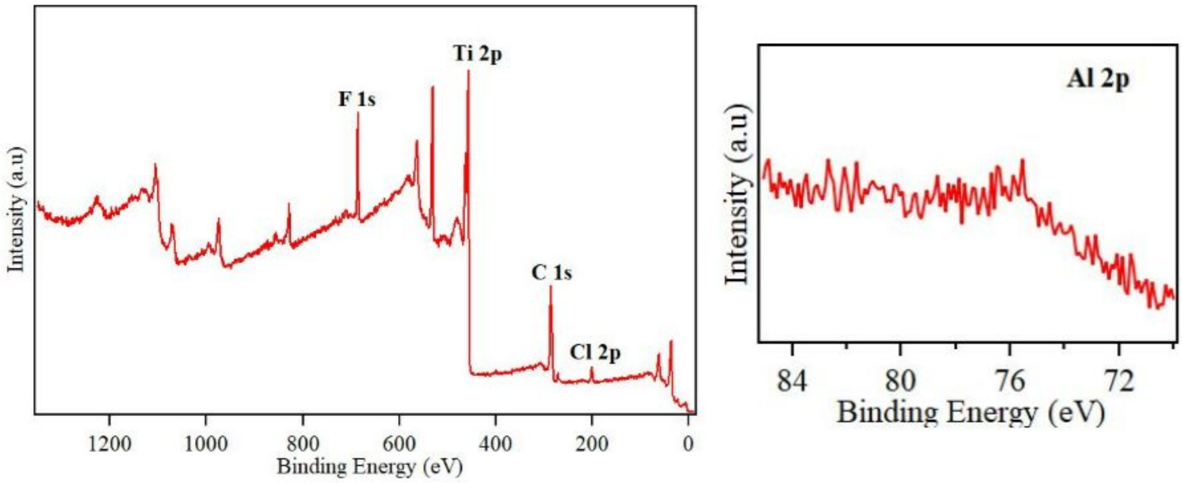

Fig.S3. XPS survey spectra of Mxene, indicating absence of Al peak

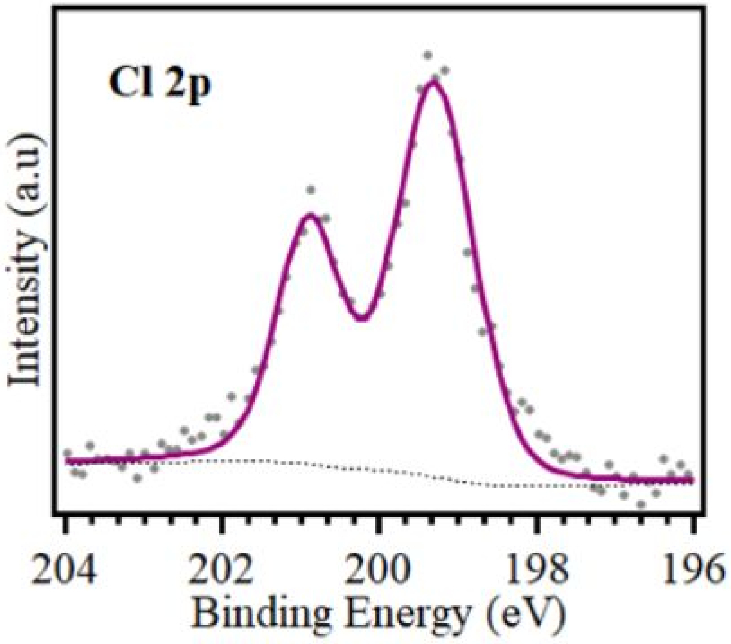

Figure S4. XPS spectra of Cl in ML MXene sample after etching

Calculation of cathode and anode mass loading:

The mass loading of each negative and positive electrodes was calculated and used out for balance the charge on both electrodes using equation,

$$\frac{m_+}{m_-} = \frac{C_- \times \Delta V_-}{C_+ \times \Delta V_+}$$

where m_- and m_+ are the mass of the active materials, C_- and C_+ are the specific capacitances, and ΔV_- and ΔV_+ are the potential windows of the anode and cathode, respectively. Calculated results for scan rate 50 mV/s are about 1.47. So, the average mass of cathode ($NaMnO_2$) was 6 mg, and anode (ML Mxene) - 4 mg. Electrodes were prepared by repeated brush coating onto nickel foam (thickness: 0.9 mm, bulk density: 0.62 g/cm^3) current collectors.

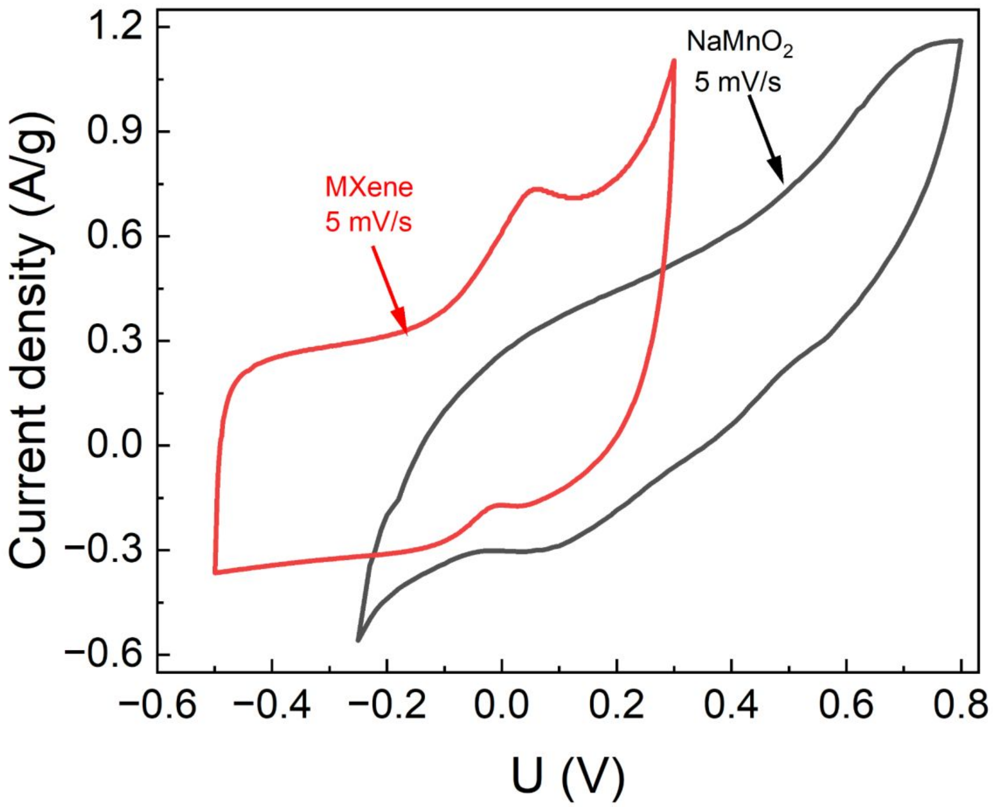

Figure S5. CVA of three electrode cells with electrodes based on $NaMnO_2$ and ML Mxene in Na_2SO_4 electrolyte.

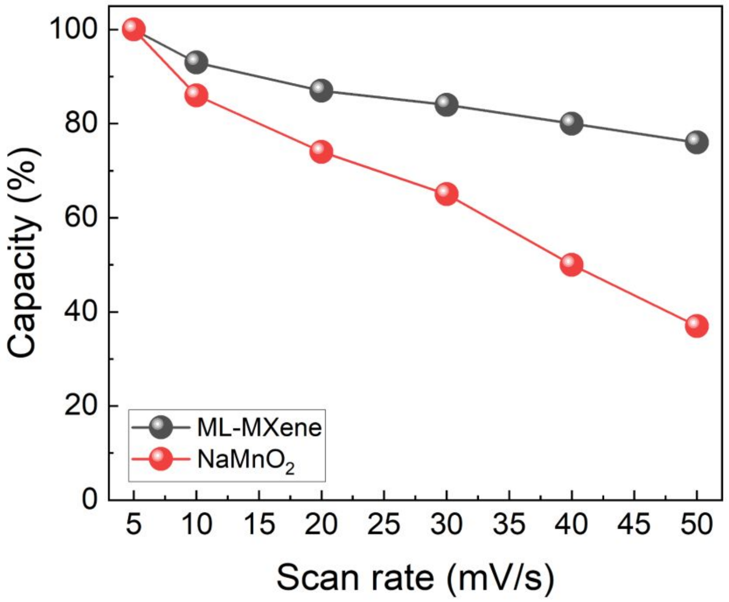

Figure S6. Capacity retention of $NaMnO_2$ and ML MXene electrodes during an increase of scan rate (from 5 mV/s to 50 mV/s) compared to the calculated capacity value at 5mV/s.

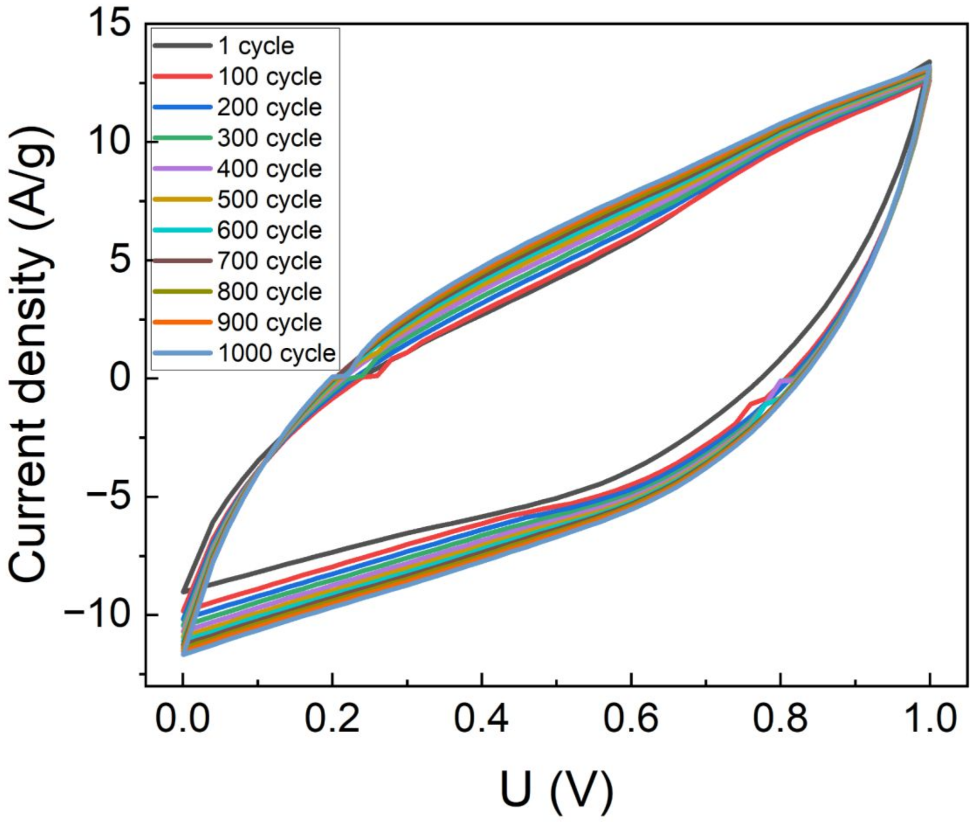

Figure S7. CVA cycling of coin cells in aqueous Na_2SO_4 electrolyte (scan rate 50 mV/s).

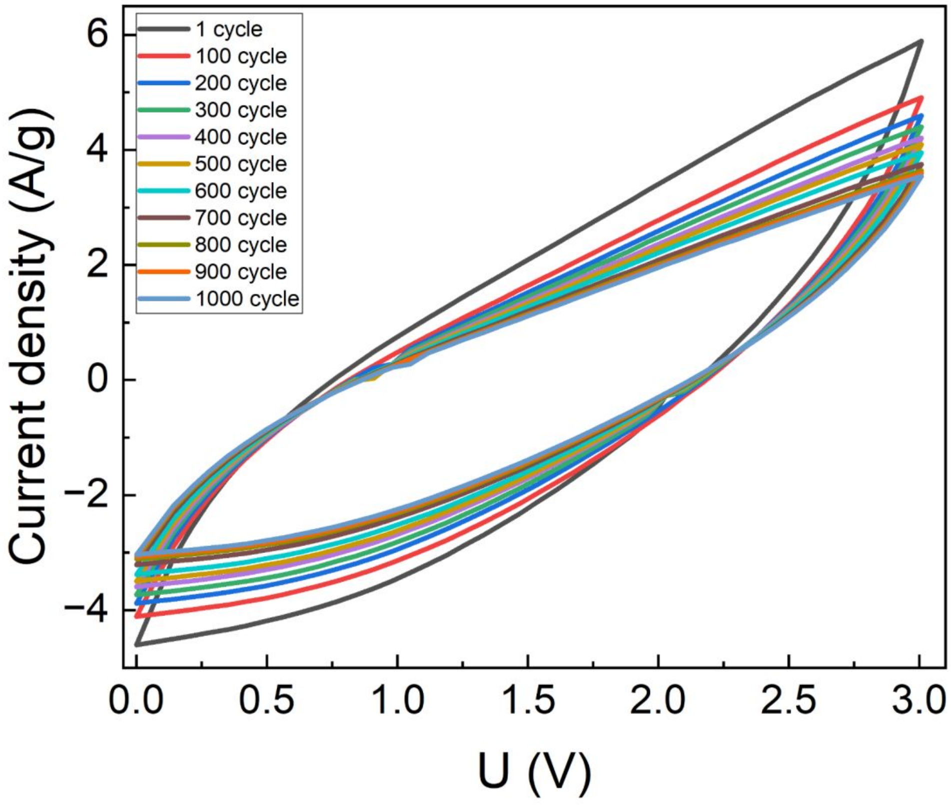

Figure S8. CVA cycling of coin cells in $NaPF_6$ electrolyte (scan rate 50 mV/s).

Properties	Aqueous hybrid system	Non-aqueous hybrid system
Electrolyte	Na_2SO_4	$NaPF_6$ in ED:DMC
Voltage window (V)	0-1	0-3
Discharge current density range (A/g)	1-20	0.5-20
Discharge time (s) (current density - 1 A/g)	150	190
Specific capacity (Ah/kg)	120 (current density 1A/g)	170 (current density 0.5A/g)
Max Energy density (Wh/kg)	90	360
Max Power density ((Wh/kg))	610	970
Capacity retention (%) after 1000 cycles	97	88
Coulombic efficiency (%) after 1000 cycles	82	98

Table ST2. Performance comparison of hybrid aqueous and non-aqueous systems

Type of system	Electrodes	Electrolytes	Mass loading	Reference
hybrid	Na-MnO_2 / $Ti_3C_2T_x$	Na_2SO_4	0.3 mg/cm^2	[47]
hybrid	MnO_2 / $Ti_3C_2T_x$	KOH	not specified	[48]
hybrid	MnO_2 / $Ti_3C_2T_x$	Na_2SO_4	8 mg/cm^2	[49]
battery	$NaMn_{0.89}Fe_{0.11}O_2$ / Na	$NaClO_4$	not specified	[50]
hybrid	MnO_2 / Ti_2C_2	Na_2SO_4	not specified	[51]
asymmetric	δ-MnO_2 / Mxene	Na_2SO_4	1.5 mg/cm^2	[52]

Table ST3. Comparison of the characteristics of reported electrode systems.
